# Damping factor in magnetorheological shear stiffening gel: Fabrication and formulation of a fractional constitutive model

**DOI:** 10.1371/journal.pone.0323432

**Published:** 2025-05-08

**Authors:** Fei Guo, Youmei Yu, Xiaoguo Lin, Chengbin Du

**Affiliations:** 1 School of Civil Engineering and Architecture, Anhui Polytechnic University, Wuhu, China; 2 Anhui Engineering Research Center of Green Building and Digital Construction, Wuhu, China; 3 School of Civil and Transportation Engineering, Ningbo University of Technology, Ningbo, China; 4 Department of Engineering Mechanics, Hohai University, Nanjing, China; National University of Sciences and Technology, PAKISTAN

## Abstract

A variety of magnetorheological shear stiffening gels (MRSSGs) were synthesised by incorporating varying amounts of carbonyl iron powder (CIP) into a shear stiffening gel (SSG) matrix. The dynamic damping performance of the MRSSG was initially evaluated using a rheometer. The damping factor of MRSSGs demonstrates magnetic-sensitive characteristics and can autonomously respond to external stimuli due to B-O cross-linked bonds. The examination of the effects of applied frequency and magnetic field on the damping factor included the delineation of viscous damping from the SSG matrix, magneto-induced damping, and interfacial damping, leading to the development of an innovative fractional constitutive model. This model explicitly illustrates the relationship among the damping factor, shear angular frequency, and magnetic field strength. Theoretical results of the damping factor, derived from modelling calculations and analyses, closely align with experimental findings as excitation angular frequencies vary across different magnetic induction intensities, exhibiting a high fitting accuracy with a correlation coefficient exceeding 0.999. Furthermore, an augmentation in magnetic induction strength correlates with a reduction in the fractional order value, which declines from 0.96 to 0.49.

## 1. Introduction

Magnetorheological shear stiffening gel (MRSSG) is a sophisticated material characterised by its multifunctionality, demonstrating both shear thickening (ST) and magnetorheological (MR) properties as a result of the uniform dispersion of micron-sized soft magnetic particles within a shear stiffening gel (SSG) matrix. The ST effect is characterised by a significant increase in modulus or viscosity when exposed to elevated external strain rates, demonstrating a distinctive phenomenon in polymer materials and fluid suspensions. Currently, ST materials provide significant potential for vibration reduction, structural buffering [1–4], and impact protection [5–9] due to their sensitive rate-dependent properties. The use of a magnetic field causes the alignment of magnetic particles in MRSSG into chains, leading to alterations in the mechanical properties of materials, known as the MR effect [10,11].

By incorporating magnetically responsive particles into the SSG matrix, SSG can attain directional modulation of mechanical characteristics in a magnetic field, akin to magnetorheological elastomers (MRE) [12]. Consequently, multi-functional composites exhibiting both rate-sensitive properties and magnetorheological effects have increasingly emerged as a focal point of research. Testa [13] developed a magnetic shape memory composite by encapsulating magnetorheological droplets within a polydimethylsiloxane (PDMS) matrix. Through embossing, basic shearing, and unrestricted three-dimensional deformation, it is demonstrated that the material exhibits rapid and entirely reversible magnetic shape memory. Borin [14] utilized PDMS as the matrix, incorporating spherical carbonyl iron particles (CIPs) and irregularly shaped NdFeB particles, and subsequently evaluated the resultant heterogeneous magnetically active elastomer. Wang [15] performed the preliminary study on the multifunctional polymer incorporating CIPs and Fe_3_O_4_ particles as additives. Yuan [16] produced a multifunctional composite that has a pre-magnetization enhancing effect by integrating NdFeB into SSG. The storage modulus of the sample augmented by a factor of 1.43 after pre-structuring with a 1200 mT magnetic field. Guo [17] created a multifunctional composite by including CIPs into Silly Putty, demonstrating rate-sensitive characteristics, and analyzed its MR and ST properties. The mechanism of the ST phenomenon has been thoroughly examined in recent years. The rate-sensitive characteristics of ST materials demonstrated the jamming phenomena, with the loading rate markedly affecting the phase transformation of ST materials [18–20]. When external stress is applied for a duration shorter than the relaxation time of the cross-linked bonds, the molecular chains demonstrate mutual attraction, hence constraining their movement and impacting the ST performance [21]. Wang [22] noted that in a Silly Putty reinforced composite, the Silly Putty successfully absorbs external stresses during knee protection tests because to the B-O bond. Liu [23] constructed a silicon-boron copolymer and analyzed the B-O-B, B-O-H, and Si-O-B bonds generated during the reaction of polydimethylsiloxane (PDMS) with boric acid using Fourier transform infrared (FTIR) spectrometry. Liu also produced a multifunctional magnetorheological gel (MMRG) and examined the correlation between the ST effect and the quantity of cross-linked bonds through experimental methods and a proposed molecular chain model [24,25]. Wang [26] created a self-healing shape memory material, illustrating that the disruption and reformation of the B-O bond is the pivotal ingredient in this process. Consequently, in consideration of the previously mentioned studies, a growing number of researchers have focused on the synthesis and chemical processes of multifunctional composites. The microscopic magneto-induced mechanical model for these composites, affected by magnetic fillers in the SSGs, has been neglected. The arrangement of magnetic particles in the composites produced a catenulate structure as a result of the magnetic field’s impact, hence enhancing the storage modulus [17]. Nonetheless, as a novel category of soft multifunctional polymer, damping performance is a mechanical feature of comparable significance to the elastic modulus. An increasing number of researchers have concentrated on the dynamic storage modulus and the magneto-induced microscopic model of the novel multifunctional material. Yang [27] developed an enhanced Bouc-Wen model utilising fractional derivatives, capable of precisely representing mechanical behaviour throughout a broad spectrum of strain amplitudes, excitation frequencies, and magnetic fields. The macroscopic constitutive model effectively characterises dynamic mechanical properties but fails to account for the magnetic interactions among particles. Deng [28] developed a mechanical model that accounts for the interaction between particles and the matrix. The Eshelby equivalent inclusion theory and the Mori-Tanaka method were employed to examine the influence of magnetic field strength, strain amplitude, frequency, and particle content on the shear modulus and damping, thereby facilitating cross-scale calculations from the micro to macro perspective. However, the damping property has been overlooked. Consequently, it is imperative to establish a suitable mechanical model to quantify the damping properties across diverse frequencies and magnetic field intensities for the multifunctional composites.

**Fig 1:**
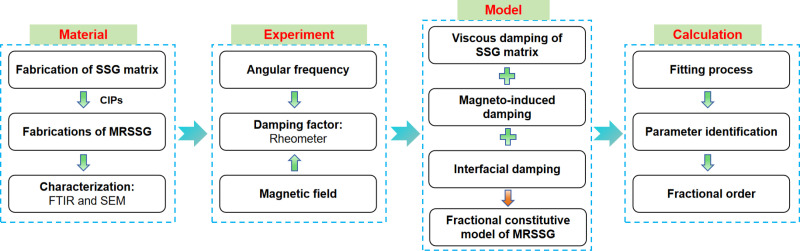
The research flowchart of the damping properties of MRSSG.

This study involved the synthesis of MRSSG samples by incorporating varying amounts of CIPs into the SSG matrix. The dynamic rheological studies demonstrated that this category of smart material displays regulated damping properties concerning angular frequency and magnetic field. A fractional constitutive model integrating viscous damping, magneto-induced damping, and interfacial damping was established to elucidate the relationship among the damping factor, shear angular frequency, and magnetic field intensity. Fig 1 illustrates the research flowchart pertaining to the damping properties of MRSSG.

## 2. Materials and methods

### 2.1. Materials

**Fig 2:**
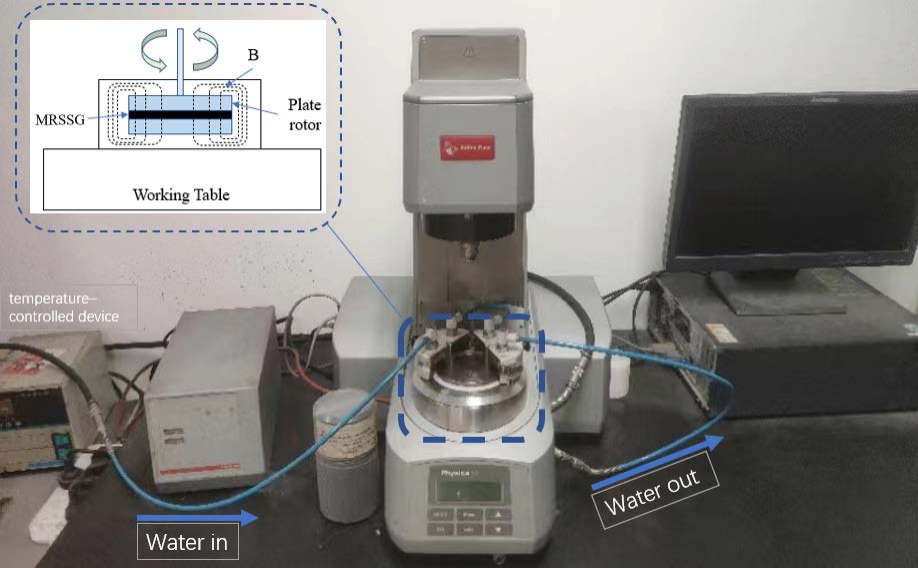
The MCR series rheometer.

The materials utilized in the synthesis of the MRSSG comprise boric acid (analytical reagent, Sinopharm Chemical Reagent Co., Ltd, Shanghai, China), PDMS (Sinopharm Chemical Reagent Co., Ltd), absolute ethyl alcohol (dispersing agent and analytical reagent, Tianjin BASF Chemical Co., Ltd, Tianjin, China), the cross-linking agent benzoyl peroxide (BPO) (Sinopharm Chemical Reagent Co., Ltd), and CIP with an average particle size of 3.5µm (Jiangsu Tianyi Ultra-fine Metal Powder Co., Ltd, Xuyi, China). The rheological properties were evaluated using an MCR series rheometer from Anton Paar Co., Austria, as depicted in Fig 2. The MRSSG sample is uniformly positioned between two parallel discs (PP20 measuring head), and the dynamic mechanical properties, including storage modulus and damping factor, were assessed in oscillatory mode utilising a sinusoidal driving force to operate the parallel-plate rotor during testing. The controllable parameters in this mode include strain amplitude, angular frequency, and magnetic field.

### 2.2. Preparation process

**Fig 3:**
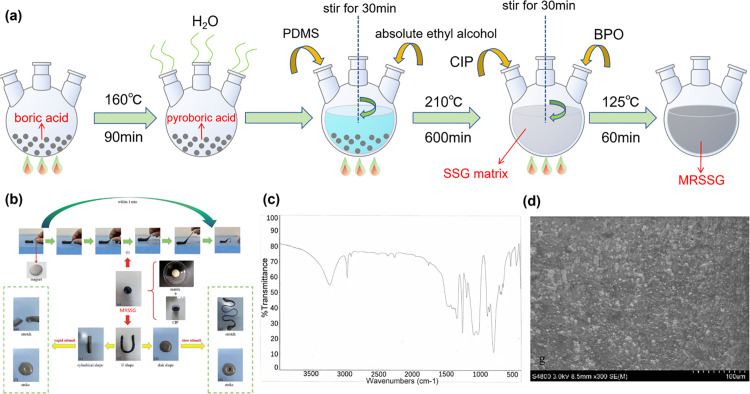
The fabrication and characterization of MRSSG. (A) The preparation procedure of the MRSSG; (B) the morphology of the material post-preparation and molding into diverse forms, as well as its reactions to rapid and low stimuli; (C) the FTIR spectroscopy spectrum spanning 4000–500 cm^^−1^^; (D) the SEM electron microscopy image illustrating the internal microstructure.

Fig 3a depicts the preparation of the MRSSG. The preliminary heating procedure led to the synthesis of pyroboric acid from boric acid, accompanied by a mass fraction decrease of roughly 30%. The second stage generated an SSG matrix demonstrating a ST effect. The concluding stage yielded the MRSSG sample after the curing procedure. The preparation steps were outlined as follows:

(1) A precise quantity of boric acid was measured and placed in a vacuum drying oven. The sample was subjected to continuous heating for 90 minutes at 160°C, resulting in the synthesis of pyroboric acid.
(2) Pyroboric acid was mixed with PDMS and absolute ethyl alcohol in a beaker. After a 30-minute stirring period, the mixture was transferred to the vacuum drying oven. The matrix was obtained after a heating duration of 600 minutes at 210°C, followed by a cooling phase.
(3) CIP and BPO were added to the matrix, followed by stirring for 30 minutes and curing at 125°Cfor 60 minutes in a vacuum drying oven. Upon cooling, the MRSSG was obtained, with the compositions and samples detailed in Table 1 and Fig 3b–3d.

**Table 1:** The compositions of the SSG matrix and MRSSG samples.

SSG matrix	wt%	MRSSG-1	wt%	MRSSG-2	wt%
PDMS	80	SSG matrix	57.8	SSG matrix	48.3
Pyroboric acid	15	CIP	40.5	CIP	50.2
Absolute ethyl alcohol	5	BPO	1.7	BPO	1.5
**MRSSG-3**	**wt%**	**MRSSG-4**	**wt%**	**MRSSG-5**	**wt%**
SSG matrix	36.1	SSG matrix	28.9	SSG matrix	22.2
CIP	62.8	CIP	70.2	CIP	77.1
BPO	1.1	BPO	0.9	BPO	0.7

Fig 3b illustrates the prepared MRSSG sample. It can be easily formed into many configurations, including cylindrical, U-shaped, or disk-shaped forms. When subjected to a sudden impulsive load or shock, the MRSSG will demonstrate stiffening behavior to resist the external force. Conversely, the application of minimal stimuli causes the material to be easily extended into a slender filament or compressed to form a profound cavity. In the presence of a magnetic field, its shape will swiftly change in accordance with the magnetic induction lines. Fig 3c displays the FTIR spectrum of the MRSSG in the range of 4000–500 cm^-1^. The absorption peak at 2950 cm^-1^ relates to the asymmetric stretching of methyl groups. The peak at 1350 cm^-1^ is ascribed to the B-O vibration. The band at 1100 cm^-1^ indicates the existence of the Si-O bond. The peaks at 890 cm^-1^ and 860 cm^-1^ are indicative of the Si-O-B bond, signifying the development of MRSSG and the existence of cross-linked bonds. Fig 3d displays the SEM picture obtained using a Hitachi S4800 scanning electron microscope. The CIP is evenly distributed throughout the matrix.

## 3. Results and discussion

### 3.1. The dynamic rheological properties of MRSSGs

**Fig 4:**
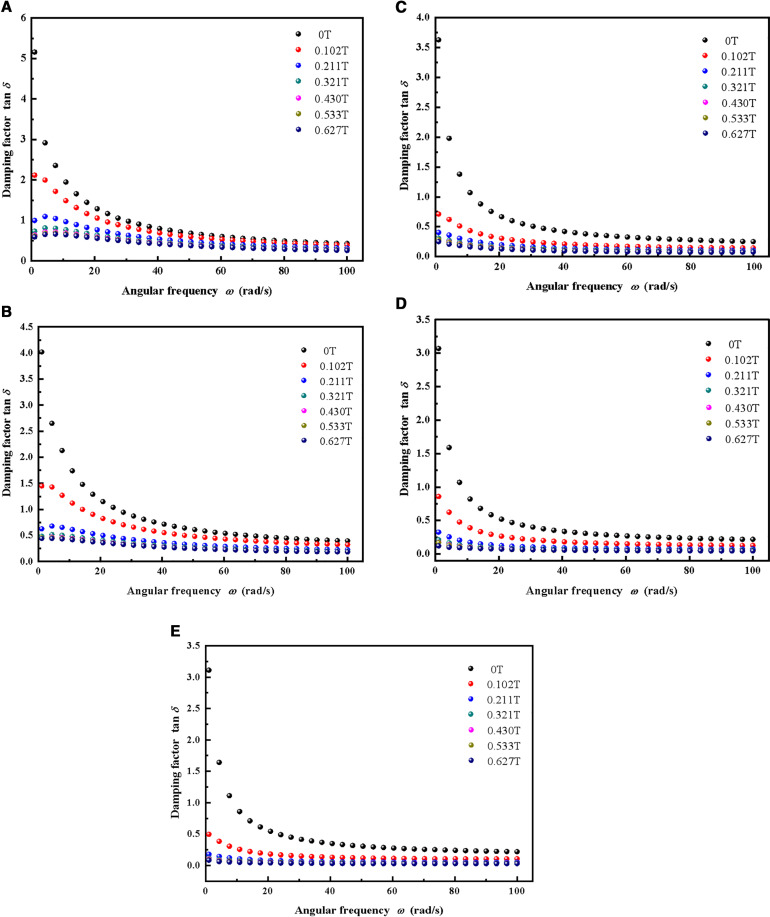
The damping factor of MRSSGs as a function of angular frequency at different magnetic flux density. (A) MRSSG-1; (B) MRSSG-2; (C) MRSSG-3; (D) MRSSG-4; (E) MRSSG-5.

Fig 4 depicts the relationship between the damping factor and the angular frequency of MRSSG samples with different SSG matrices at various magnetic induction intensities. The angular frequency is systematically altered from 1 to 100 rad/s, maintaining a constant shear strain value of 0.1%. In the absence of an external magnetic field, the damping performance of each sample group is ideal, with the damping factor varying significantly from 5.160 to 0.494 as frequency changes. In comparison to the prior silicone rubber-based MRE [29], the MRSSG demonstrates a more pronounced rate sensitivity and damping performance. Moreover, the damping factor of each sample group progressively stabilises once the loading angular frequency attains 40 rad/s, with all values remaining below 1.0. Concurrently, when the intensity of magnetic induction escalates from 0T to 0.627T, the damping factor of the sample stays predominantly invariant to fluctuations in frequency. In other words, at elevated magnetic field intensities, the damping factor is mostly unaffected by frequency. On one hand, the soft magnetic particles within MRSSGs are magnetized by the magnetic field, generating and transferring magnetic interaction forces to the matrix, whose modulus is significantly lower than that of the soft magnetic particles, making it susceptible to deformation. If the magnetic field is weak, the contact force among the particles is minimal, resulting in a proportionally little force transmitted to the matrix. Currently, the soft magnetic particles and the matrix are somewhat unconstrained, resulting in significant slippage and increased damping. A strong applied magnetic field enhances the force between the particle and the matrix, impeding their relative motion, which leads to a reduction in slip displacement and less damping. Conversely, an elevation in the concentration of soft magnetic particles leads to an augmented volume fraction of particles and a diminished volume fraction of the matrix. Given that the intrinsic damping of soft magnetic particles is considerably lower than that of matrix materials, the damping of composite materials decreases as the concentration of soft magnetic particles increases. For instance, when the CIP content rises to 77.1% (MRSSG-5), without an external magnetic field, the peak damping factor at an angular frequency of 1 rad/s decreases to 3.11.

**Fig 5:**
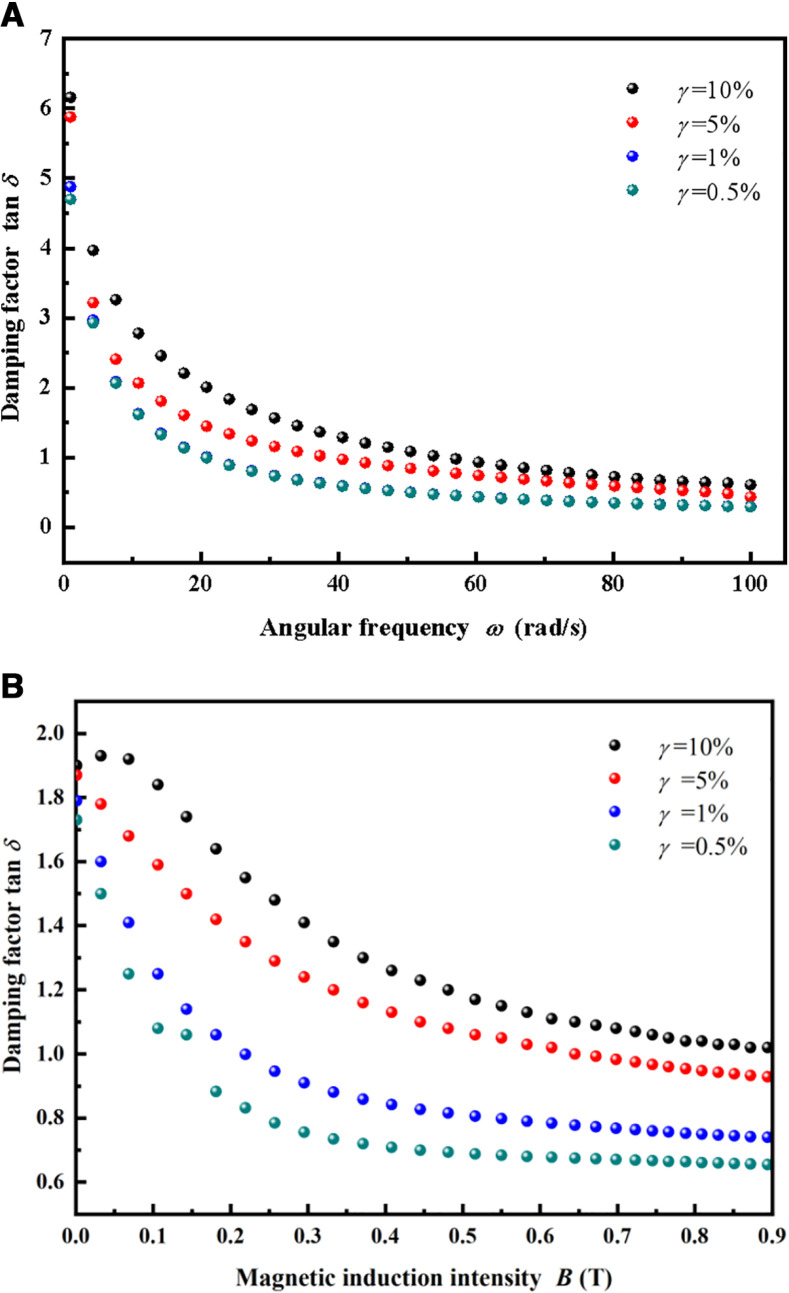
The effect of shear strain on damping factor (MRSSG-2). (A) Damping factor with angular frequency in the absence of magnetic field; (B) damping factor with magnetic induction intensity at angular frequency of 20 rad/s.

Fig 5 illustrates the relationships between the damping factor and angular frequency, as well as the damping factor and magnetic induction intensity of sample MRSSG-2 at varying shear strain amplitudes of 0.5%, 1%, 5%, and 10%. The angular frequency is varied continuously from 1 to 100 rad/s, while the magnetic induction intensity is varied continuously from 0 to 0.894 T. As the applied strain escalates, the deformation of the matrix material intensifies, leading to increased relative slip between the particles and the matrix, so enhancing the damping effect. When the MRSSG-2 sample experiences a significant shear strain of 10% in the absence of a magnetic field, the damping factor may reach 6.16. Conversely, decreasing applied shear strains lead to reduced relative slip and hence, decreased damping. Similarly, although the strength of magnetic induction increases at a constant angular frequency of 20 rad/s, the damping factor gradually decreases, but the effect of shear strain on the damping factor stays same.

### 3.2. Fractional constitutive model for damping factor

**Fig 6:**
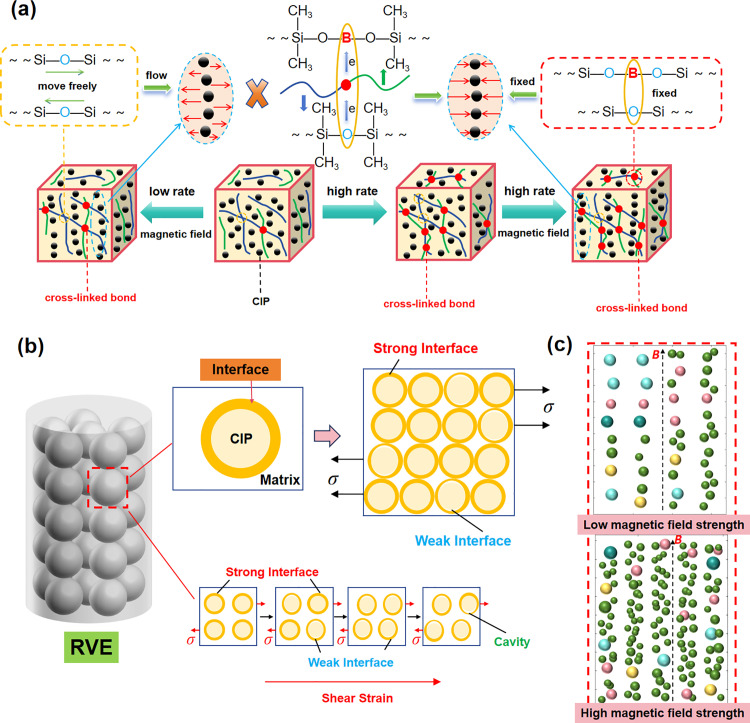
The working mechanism of MRSSG. (A) Mechanism of CIP particle chain formation in MRSSG with varying molecular chain dynamics; (B) interface working mechanism; (C) different ordered structures of CIP particles under low and high magnetic field strength respectively.

The viscosity of the SSG matrix is markedly greater than that of the oily carrier fluid, demonstrating pronounced rate sensitivity features. The electron-deficient p orbital of the boron atom could acquire electrons from the oxygen in the silicon-oxygen combination. Subsequently, the breakdown of BPO would link the molecular chains, resulting in the formation of B-O cross-linked bonds [15]. At heightened levels of external stimulation, the internal B-O cross-linked bonds may impede the relative motion of individual chains, leading to the development of a network structure from the linear molecular chains. During the development of CIP particle chains in the presence of a magnetic field, the CIPs are prone to immobilization due to the effect of the cross-linked molecule chains, as depicted in Fig 6a. Nevertheless, when the external excitation rate is minimal, only a restricted quantity of B-O cross-linked bonds is established, permitting a greater number of individual Si-O-Si chains to move unimpeded.

Besides exhibiting changing stiffness, MRSSG also induces corresponding variations in damping when the frequency and magnetic field are adjusted. Material damping is typically considered to arise from matrix viscous damping, magneto-induced damping, and interfacial damping. The examination of deformation characteristics, especially the response to dynamic loads, is crucial for damping materials. The SSG matrix demonstrates pronounced viscoelasticity, characterized by reversible elastic deformation and time-dependent viscous deformation. The irreversible deformation of molecular chain segments within the matrix necessitates a specific duration for the macromolecular conformation to attain a new equilibrium in response to external pressures, a phenomenon known as “hysteresis”. Each cycle experiences energy loss, referred to as “internal consumption”, where the labor expended is wasted as thermal energy. Increased internal consumption results in increased absorption of vibrational energy, thereby enhancing the damping effect. The process of magneto-induced damping is believed to occur mainly by the absorption of external energy to counteract the magnetic interactions among adjacent particles, namely the transformation of elastic energy into magnetic energy, followed by its dissipation through hysteresis loss. Fig 6c illustrates that CIP particles can develop various ordered structures in response to low and high magnetic field strengths, with increased vibrational energy absorption resulting in greater damping [17]. Interfacial damping, arising from the interaction between the particles and the matrix, considerably influences the material’s overall damping properties. An intermediate interfacial phase is formed when the molecular structure of the matrix adheres to the particle surface. This phase predominantly facilitates the passage of stress between the particles and the matrix. Upon the application of significant strain, energy must be absorbed to facilitate the viscous flow of material adjacent to the particle interface and to disrupt the adhesion between the particles and the matrix. This process results in the transformation of elastic energy into kinetic energy, which is subsequently converted into thermal energy, thereby producing a damping effect. In accordance with the micro-mechanical theory of composites, a representative volume element (RVE) is chosen to depict the average stress-strain response of the MRSSG, as seen in Fig 6b. The RVE and MRSSG exhibit identical macroscopic properties, including modulus and damping. Nevertheless, an optimal interface is unattainable, as an incomplete amalgamation of particles and matrix is inevitable during the manufacture of MRSSG. In the absence of a coating on the particles, the occurrence of cavities is quite probable. The impact of cavities is beyond the research scope of this study, as it concentrates on the properties of MRSSG devices under standard operating settings. Under this state, the MRSSG experiences varied stress and is devoid of cavities. The presence of cavities indicates that the tension on the MRSSG exceeds its operational limits or that the MRSSG is nearing fatigue. The interface’s strength is expected to vary with escalating external shear stress. When minimal stress is applied to the RVE, the particles stay unaltered, and the contact exhibits robust adhesion. As stress progressively escalates to a critical resolved shear stress (CRSS), certain interfaces inside the RVE begin to exhibit weak bonding. Cavities between particles and the matrix will form if the shear stress is sufficiently high. Consequently, the total dampening of the MRSSG can be articulated as


$$\kappa_{MRSSG}=\kappa_{V}+\kappa_{M}+\kappa_{I}=\tan\delta$$ (1)

where *κ*_*MRSSG*_ is the overall damping of the material, *κ*_*V*_ is the matrix viscous damping, *κ*_*M*_ is the magneto-induced damping, and*κ*_*I*_ is the interfacial damping. The damping factor tan*δ* is the main index to measure the damping characteristics, therefore, the damping factor is used as the parameter to quantify the damping of the material in this section.

#### 3.2.1. Viscous damping of SSG matrix.

MRSSG demonstrates pronounced rate-sensitive characteristics, with frequency significantly influencing its dynamic mechanical properties, indicating a robust time dependence. Conversely, the conventional viscoelastic mechanical model, which integrates the Hookean spring and the traditional Newtonian dashpot, fails to accurately represent the mechanical behavior of viscoelastic materials in accordance with experimental data. The introduction of the fractional order derivative model significantly addresses the shortcomings of the traditional viscoelastic model.

Fractional order derivatives are presently characterized by diverse definitions and lack a standardized form. This section employs the prevalent Riemann-Liouville type fractional order operator [30], defined under the assumption that the time function *f*(*t*) equals zero. At *t* = 0, the Riemann-Liouville fractional-order integrals are


$$D^{-\alpha }f(t)=\frac{{\text{d}}^{-\alpha }f(t)}{{\text{d}}^{-\alpha }t}=\frac{1}{\Gamma (\alpha )}\int_{0}^{t}{\left(t-\tau \right)}^{\alpha -1}f(\tau )\text{d}\tau ,\;\;\;\;\;\;\;\;\;\;\;\;t>0,\;\;\;\;\alpha \in R^{+}$$ (2)

where is the gamma function and the negative sign before the fractional order denotes the integral, then the Riemann-Liouville type *α* order fractional derivative is


$$\begin{array}{cc} & D^{\alpha }f(t)=\frac{{\text{d}}^{\alpha }f(t)}{{\text{d}}^{\alpha }t}=\frac{{\text{d}}^{n}}{{\text{d}}^{n}t}[D^{-(n-\alpha )}f(t)]=\frac{{\text{d}}^{n}}{{\text{d}}^{n}t}[\int_{0}^{t}\frac{{\left(t-\tau \right)}^{n-\alpha -1}}{\Gamma (n-\alpha )}f(\tau )\text{d}\tau ]\\ & =\frac{{\text{d}}^{n}}{{\text{d}}^{n}t}[\int_{0}^{t}\frac{\tau^{n-\alpha -1}}{\Gamma (n-\alpha )}f(t-\tau )\text{d}\tau ] \end{array}$$ (3)

whereα>0 andn−1≤α≤n, *n* are positive integers.

If *n* = 1, Eq. (3) can be changed to


$$D^{\alpha }f(t)=\frac{\text{d}}{\text{d}t}\int_{0}^{t}\frac{\tau^{-\alpha }}{\Gamma (1-\alpha )}f(t-\tau )\text{d}\tau$$ (4)

The Fourier transform with respect to the fractional order derivative is as follows


$$F[D^{\alpha }f(t)]={\left(\text{i}\omega \right)}^{\alpha }f(\omega )$$ (5)

*ω* is the angular frequency, the transform enables conversion from the time domain to the frequency domain.

**Fig 7:**
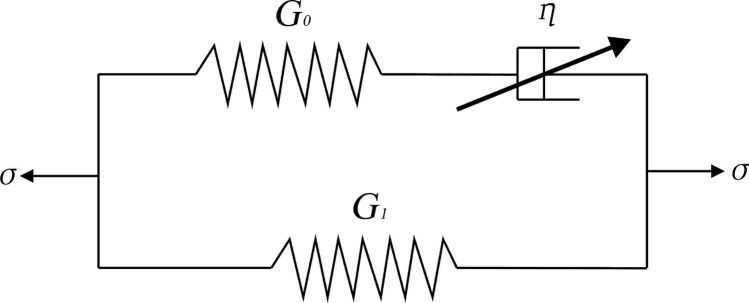
Fractional Poynting-Thomson model (FPT).

In this section, based on the classical Poynting-Thomson model [31], a fractional Poynting-Thomson model (FPT) is constructed by introducing the fractional order differential operator, as shown in Fig 7.

The components *G*_0_ and *G*_1_ are the modulus of elasticity of the Hooke spring; *η* is the viscosity coefficient of the Abel dashpot with fractional operator, and its stress-strain constitutive relation [32] can be expressed as$\sigma =\eta D^{\alpha }[\varepsilon (t)]$, and whenα=1_,_ the Abel dashpot reduces to a conventional Newtonian dashpot. In general, the parameter value of *α* in the Abel dashpot is0<α<1.

The constitutive relationship for the FPT model can be introduced as


$$\sigma (t)=\frac{G_{0}G_{1}+(G_{0}+G_{1})\eta D^{\alpha (t)}}{G_{0}+\eta D^{\alpha (t)}}\varepsilon (t)$$ (6)

Fourier transform of Eq. (6) using fractional order differentials


$$\sigma (\omega )=\frac{G_{0}G_{1}+(G_{0}+G_{1})\eta {\left(\text{i}\omega \right)}^{\alpha }}{G_{0}+\eta {\left(\text{i}\omega \right)}^{\alpha }}\varepsilon (\omega )$$ (7)

From the definition of complex modulus, the SSG matrix complex modulus is obtained as


$$Y^{*}(\omega )=\frac{\sigma (\omega )}{\varepsilon (\omega )}=\frac{G_{0}G_{1}+(G_{0}+G_{1})\eta {\left(\text{i}\omega \right)}^{\alpha }}{G_{0}+\eta {\left(\text{i}\omega \right)}^{\alpha }}=Y_{1}(\omega )+\text{i}Y_{2}(\omega )$$ (8)

Due to${\text{i}}^{\alpha }=\cos\frac{\alpha \pi }{2}+\text{i}\sin\frac{\alpha \pi }{2}$ [33], the energy storage modulus *Y*_1_(*ω*) and loss modulus *Y*_2_(*ω*) of the substrate can be obtained as


$$Y_{1}(\omega )=G_{1}+\frac{\eta^{2}\omega^{2\alpha }G_{0}+\eta \omega^{\alpha }{G_{0}}^{2}\cos(\alpha \pi /2)}{{G_{0}}^{2}+\eta^{2}\omega^{2\alpha }+2\eta \omega^{\alpha }G_{0}\cos(\alpha \pi /2)}$$ (9)

$$Y_{2}(\omega )=\frac{\eta \omega^{\alpha }{G_{0}}^{2}\sin(\alpha \pi /2)}{{G_{0}}^{2}+\eta^{2}\omega^{2\alpha }+2\eta \omega^{\alpha }G_{0}\cos(\alpha \pi /2)}$$ (10)

According to the definition of damping factor, the viscous damping of SSG matrix based on FPT model can be expressed as


$$\kappa_{V}=\frac{Y_{2}(\omega )}{Y_{1}(\omega )}=\frac{\eta \omega^{\alpha }{G_{0}}^{2}\sin(\alpha \pi /2)}{{G_{0}}^{2}G_{1}+\eta^{2}\omega^{2\alpha }(G_{0}+G_{1})+\eta \omega^{\alpha }G_{0}(G_{0}+2G_{1})\cos(\alpha \pi /2)}$$ (11)

It defines the ratio of energy expended every vibration cycle to the maximal strain energy.

#### 3.2.2. Magneto-induced damping.

Upon the application of an external magnetic field, the soft magnetic particles within the matrix become magnetized, resulting in magnetic interactions among neighboring ferromagnetic particles in the chain. During shear strain, a fraction of the elastic energy must be transformed into magnetic energy to facilitate the separation of adjacent particles. Thus, it can be inferred that the magnetic field induces energy dissipation of [34].


$$E_{dissipation}=\frac{\phi^{2}J_{p}^{2}(2-\gamma^{2)}}{8\pi^{2}\mu_{0}\mu_{1}{\left(1+\gamma^{2}\right)}^{5/2}}$$ (12)

where, *ϕ* is the particle volume fraction, *γ* is the shear strain, *μ*_0_ is the vacuum permeability, *μ*_1_ is the permeability of non-conducting matrix, which is usually taken as 1. The relationship between the magnetic polarization *J*_*p*_ and the magnetization intensity *M* is *J*_*p*_* *= *μ*_0_ M.

According to the Frohlich-Kennely formula, Eq. (12) can be expressed as


$$E_{dissipation}=\frac{\mu_{0}\phi^{2}(2-\gamma^{2}){\left(\mu^{0}-1\right)}^{2}M_{s}^{2}H^{2}}{8\pi^{2}\mu_{1}{\left(1+\gamma^{2}\right)}^{5/2}{\left[M_{s}+(\mu^{0}-1)H\right]}^{2}}$$ (13)

And the elastic energy of a material can be expressed as


$$E_{elastic}=\frac{1}{2}G^{\prime }\gamma^{2}$$ (14)

where *G*^’^ is the shear energy storage modulus of the material and *µ*^0^ is the initial permeability.

Similarly, based on the definition of the damping factor, combining Eqs. (13) and (14), the magneto-induced damping expression is given by


$$\kappa_{M}=\frac{E_{dissipation}}{E_{elastic}}=\frac{\mu_{0}\phi^{2}(2-\gamma^{2}){\left(\mu^{0}-1\right)}^{2}M_{s}^{2}H^{2}}{4\pi^{2}\mu_{1}G^{\prime }\gamma^{2}{\left(1+\gamma^{2}\right)}^{5/2}{\left[M_{s}+(\mu^{0}-1)H\right]}^{2}}$$ (15)

From Eq. (13), it can be seen that the magneto-induced damping is closely related to the particle volume fraction, the applied magnetic field strength, the applied shear strain, and the saturation magnetization *M*_*s*_ of the soft magnetic particles.

#### 3.2.3. Interfacial damping.

**Fig 8:**
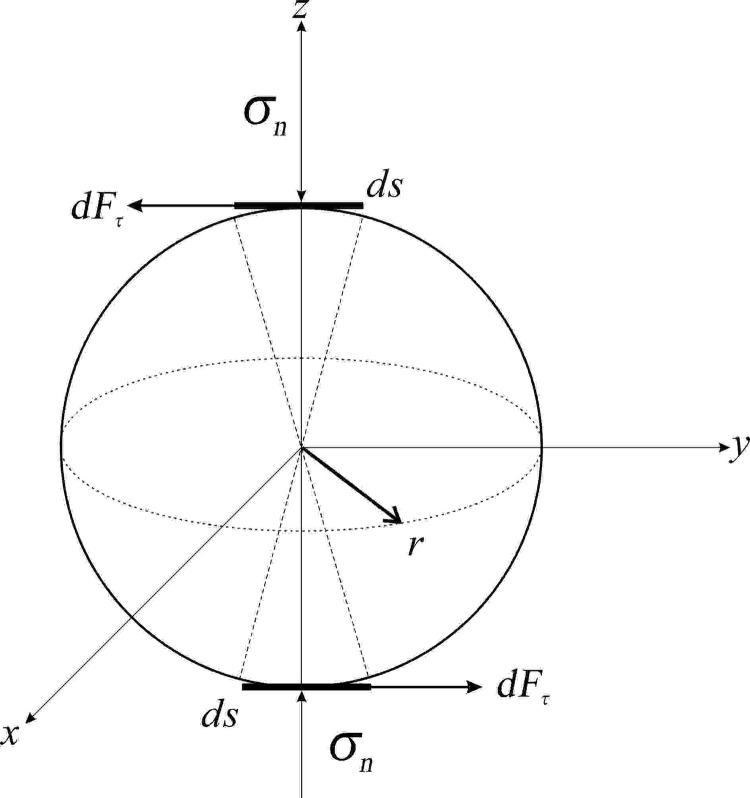
The force at the interface where relative movement is likely to occur.

When the adhesion between the soft magnetic particles and the matrix is inadequate, the interfacial damping primarily arises from the friction at the interface during material deformation, which can be characterized using Lavernia analysis [35], as illustrated in Fig 8. The research indicates that damping is primarily influenced by the friction coefficient between the particles and the matrix, along with the interfacial normal force at the potential site of relative motion.

*ds* is the displacement microelement at the location where the relative motion is generated, and *σ*_*n*_ is the normal force; when the externally applied shear stress is sufficiently large to generate the relative displacement, *r* (*γ*_0_-*γ*_crt_), where *γ*_crt_ is the critical shear strain at the time of generating the relative motion, and *γ*_0_ is the corresponding shear strain, the friction force at *ds* can be expressed as


$$dF_{\tau }=(f\sigma_{n})ds$$ (16)

where *f* is the friction coefficient between the particles and the matrix, the corresponding energy dissipation can be expressed as


$$dE_{dissipation}=r(\gamma_{0}-\gamma_{crt})(f\sigma_{n})ds$$ (17)

The energy dissipation per unit volume produced by all the particles inside the material is


$$E_{dissipation}=\frac{\sum_{i=1}^{n}\oint r(\gamma_{0}-\gamma_{crt})(f\sigma_{n})ds}{V}=\frac{3\pi }{4}f\phi \sigma_{n}(\gamma_{0}-\gamma_{crt})$$ (18)

where *V* is the volume of the material and the particle volume fraction$\phi =\frac{1}{V}\sum_{i=1}^{n}V_{i}$, *V*_i_ is the volume of the i_th_ spherical particle.

And the elasticity of material storage can provide


$$E_{elastic}=\frac{\tau_{0}^{2}}{2G^{\prime }}$$ (19)

where *τ*_0_ is the applied shear stress and *G*^’^ is the shear energy storage modulus of the material. Then, from Eqs. (18) and (19), the interfacial damping can be expressed as


$${\kappa_{I}}^{w}=\frac{E_{dissipation}}{E_{elastic}}=\frac{3\pi }{2}\cdot \frac{f\phi \sigma_{n}G^{\prime }(\gamma_{0}-\gamma_{crt})}{\tau_{0}^{2}}$$ (20)

Since *γ*_crt_ is much smaller than *γ*_0_, Eq. (20) can be simplified as


$${\kappa_{I}}^{w}=\frac{3\pi }{2}\cdot \frac{f\phi \sigma_{n}}{\tau_{0}}$$ (21)

From Fig 8, *τ*_0_ can be equated to the effective stress *σ*_*xy*_ at the particle interface in the xy-plane [36,37], i.e., *τ*_0_=*σ*_*xy*_. Eq. (21) is simplified by making *K *= *σ*_*n*_/*σ*_*xy*_, where *K* is the coefficient of concentration of the normal force at the point where the particles undergo relative motion. In addition, the deformation process caused by the relative motion at the interface through the non-uniform strain state of the material will only occur in part of the interface, considering that the relative motion produces interfacial damping, so the introduction of the correlation coefficient *C* in Eq. (21), then there are


$${\kappa_{I}}^{w}=\frac{3\pi }{2}\cdot KCf\phi$$ (22)

In contrast, when the bond between the soft magnetic particles and the matrix is strong, the frictional dissipative damping mechanism is no longer applicable and Schoeck theory [38] is used instead to characterise the interfacial damping *κ*_*I*_^*s*^. Therefore, the total interfacial damping can be expressed as


$$\kappa_{I}={\kappa_{I}}^{w}+{\kappa_{I}}^{s}=\varphi \frac{3\pi }{2}\cdot KCf\phi +(1-\varphi ){\kappa_{I}}^{s}=\varphi (\frac{3\pi }{2}\cdot KCf\phi -{\kappa_{I}}^{s})+{\kappa_{I}}^{s}$$ (23)

For MRSSG formed by filling SSG matrix with ferromagnetic particles, the contribution of the strong bonding between the particles and the matrix to the interfacial damping can be neglected, whereas according to the related study [39], the expression for the scaling factor *φ* is as follows


$$\varphi ={\left(1-\phi \right)}^{1/3}{\left(1-\gamma \right)}^{1/3}$$ (24)

From Eqs (23) and (24), the interfacial damping can be finally expressed as


$$\kappa_{I}=\frac{3\pi }{2}\cdot KCf\phi {\left(1-\phi \right)}^{1/3}{\left(1-\gamma \right)}^{1/3}$$ (25)

After the above analysis, the respective expressions for matrix viscous damping, magnetogenic damping and interfacial damping are derived, and the overall damping of MRSSG can be derived by substituting Eqs. (11), (15) and (25) into Eq. (1) as


$$\begin{array}{cc} & \kappa_{MRSSG}=\tan\delta =\frac{\eta \omega^{\alpha }{G_{0}}^{2}\sin(\alpha \pi /2)}{{G_{0}}^{2}G_{1}+\eta^{2}\omega^{2\alpha }(G_{0}+G_{1})+\eta \omega^{\alpha }G_{0}(G_{0}+2G_{1})\cos(\alpha \pi /2)}+\\ & \frac{\mu_{0}\phi^{2}(2-\gamma^{2}){\left(\mu^{0}-1\right)}^{2}M_{s}^{2}H^{2}}{4\pi^{2}\mu_{1}G^{\prime }\gamma^{2}{\left(1+\gamma^{2}\right)}^{5/2}{\left[M_{s}+(\mu^{0}-1)H\right]}^{2}}+\frac{3\pi }{2}\cdot KCf\phi {\left(1-\phi \right)}^{1/3}{\left(1-\gamma \right)}^{1/3} \end{array}$$ (26)

Eq. (26) indicates that the amplitude of the damping factor of MRSSG is intricately linked to the angular frequency, particle volume fraction, shear strain, applied magnetic field strength, and saturation magnetization.

#### 3.2.4. Model results and analysis.

**Fig 9:**
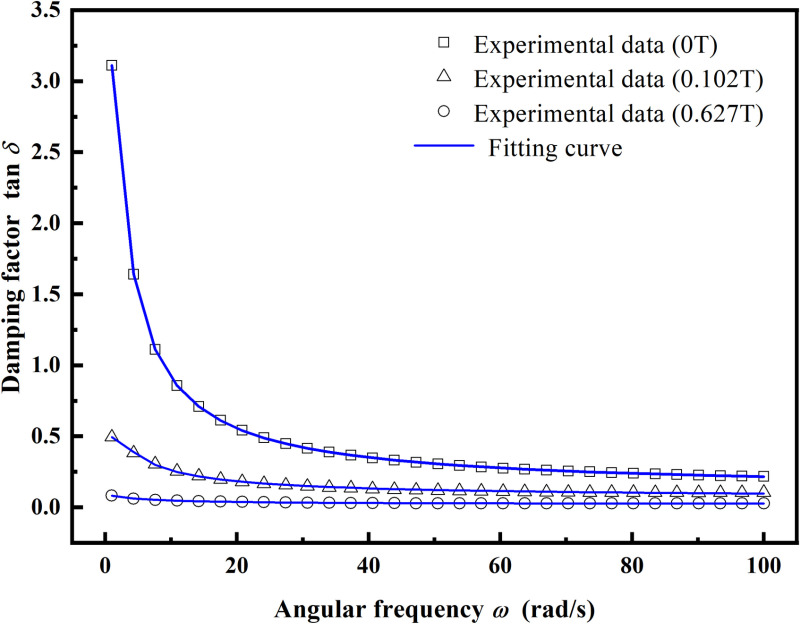
The relationship between damping factor and angular frequency for MRSSG-5.

MRSSG-5 was employed as a case study to compare its experimental data with the model for accuracy validation. Furthermore, the 1stOpt program was utilised during the fitting procedure. The model indicates that the correlation coefficient *C* is set at 0.5 [35], signifying that 50% of the interface experiences the critical strain of relative motion during deformation; the friction coefficient *f* between the soft magnetic particles and the matrix is established at 0.21 [40], and the stress concentration factor *K* at the interface is 1.2 [35]. The saturation magnetization is 1380 kA/m, while the initial permeability *µ*^0^ is 132 [41]. The permeability of the non-conducting matrix is unity. By correlating the damping factor with angular frequency of MRSSG-5 in zero-field conditions, *G*_0_ = 1.71MPa, *G*_1_ = 0.03MPa, and *η*=0.23MPa are derived. Substituting these values into Eq. (26) yields the damping factor against angular frequency of the model at varying magnetic induction strengths, as illustrated in Fig 9.

**Fig 10:**
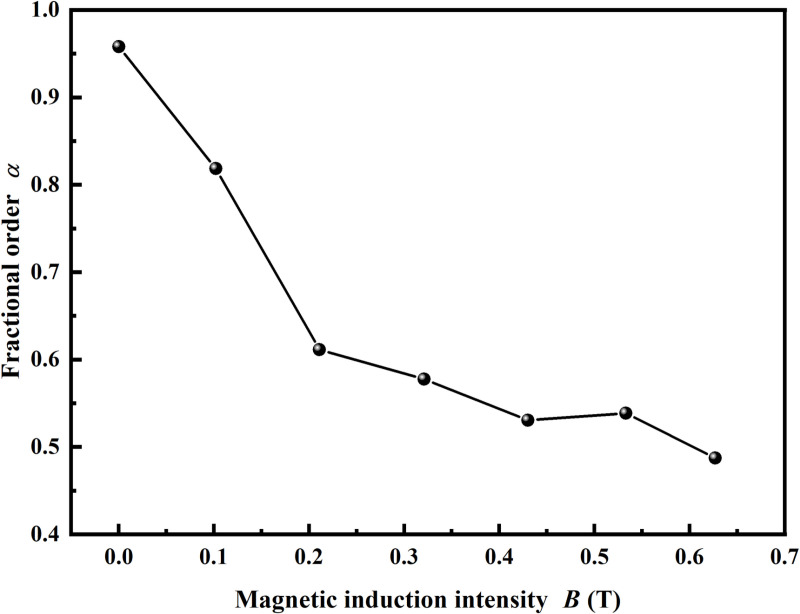
The relationship between fractional order and magnetic induction intensity for MRSSG-5.

Fig 9 demonstrates that the model closely corresponds with the experimental data, with the correlation coefficient for MRSSG-5 across various magnetic field conditions exceeding 0.999, indicating exceptional fitting accuracy. The frequency and magnetic induction strength augment the shear storage modulus of MRSSG but do not positively influence the material’s damping factor, thereby illustrating the relationship between modulus and damping as a dichotomy of opposites. The examination of the experimental data demonstrates the relationship between fractional order *α* and magnetic induction intensity, as shown in Fig 10, where a rise in magnetic induction intensity is associated with a reduction in fractional order value from 0.96 to 0.49. In the Abel dashpot, the parameter value of fractional order *α* is usually0<α<1, and when *α*=1, the Abel dashpot is simplified to the traditional Newtonian dashpot; when *α*=0, the Abel dashpot is degraded to the Hooke spring; when the magnetic induction strength increases gradually, the value of fractional order *α* decreases, and the closer the Abel dashpot is to the Hooke spring, i.e., the component of modulus of elasticity increases gradually, and the component of viscous damping decreases, which is consistent with the conclusion drawn from the experimental results.

## 4. Conclusions

This study involved the synthesis of MRSSG samples by incorporating varying concentrations of CIPs into the SSG matrix to achieve both magnetic-sensitive and rate-sensitive properties of the damping factor. A fractional constitutive model was formulated and computed for comparison with the dynamic experimental outcomes of the damping factor.

(1) Dynamic rheological experiments of MRSSGs demonstrate that CIP content, magnetic induction intensity, and angular frequency are critical factors affecting the overall damping factor. Furthermore, in the absence of a magnetic field and with a shear strain amplitude of 0.1%, the maximum damping factor for MRSSG with a CIP content of 40.5% can attain 5.16.
(2) A fractional Poynting-Thomson model was introduced to characterise the viscous damping of the SSG matrix, focussing on the influence of angular frequency and magnetic field on the damping factor for MRSSG, which is ascribed to the rate-sensitive characteristics of the matrix. The combination of magneto-induced damping and interfacial damping led to the development and validation of the fractional constitutive model.
(3) The computed modelling results of the damping factor for MRSSG, derived from varying angular frequencies at distinct magnetic induction intensities, closely align with experimental data. The fitting accuracy, indicated by a correlation coefficient exceeding 0.999, confirms the validity and suitability of the proposed model for characterising rate-sensitive materials. An rise in magnetic induction strength correlates with a reduction in the fractional order value, which declines from 0.96 to 0.49.
(4) MRSSG, a novel smart material possessing both magnetic-sensitive and rate-sensitive properties, holds significant potential for applications in semi-active structural vibration control, intelligent protection, and noise reduction. Nevertheless, to date, few smart devices utilising MRSSG have been developed and manufactured. Future research will focus on the development of intelligent damping energy dissipation structures based on MRSSG to mitigate the effects of various types of vibrations.

## Supporting information

S1 Data Set Raw data supporting the results shown in Figs 4, 5, 9 and 10. (XLSX)
